# Associations between the artificial intelligence scoring system and live birth outcomes in preimplantation genetic testing for aneuploidy cycles

**DOI:** 10.1186/s12958-024-01185-y

**Published:** 2024-01-17

**Authors:** Chun-I Lee, Chun-Chia Huang, Tsung-Hsien Lee, Hsiu-Hui Chen, En-Hui Cheng, Pin-Yao Lin, Tzu-Ning Yu, Chung-I Chen, Chien-Hong Chen, Maw-Sheng Lee

**Affiliations:** 1 Division of Infertility, Lee Women’s Hospital, Taichung, Taiwan; 2 Department of Obstetrics and Gynecology, Chung Shan Medical University Hospital, Taichung, Taiwan; 3 Institute of Medicine, Chung Shan Medical University, Taichung, Taiwan; 4 Department of Post-Baccalaureate Medicine, National Chung Hsing University, Taichung, Taiwan

**Keywords:** iDAScore, Artificial intelligence, Live birth, Single embryo transfer, Preimplantation genetic testing for aneuploidy

## Abstract

**Background:**

Several studies have demonstrated that iDAScore is more accurate in predicting pregnancy outcomes in cycles without preimplantation genetic testing for aneuploidy (PGT-A) compared to KIDScore and the Gardner criteria. However, the effectiveness of iDAScore in cycles with PGT-A has not been thoroughly investigated. Therefore, this study aims to assess the association between artificial intelligence (AI)-based iDAScore (version 1.0) and pregnancy outcomes in single-embryo transfer (SET) cycles with PGT-A.

**Methods:**

This retrospective study was approved by the Institutional Review Board of Chung Sun Medical University, Taichung, Taiwan. Patients undergoing SET cycles (*n* = 482) following PGT-A at a single reproductive center between January 2017 and June 2021. The blastocyst morphology and morphokinetics of all embryos were evaluated using a time-lapse system. The blastocysts were ranked based on the scores generated by iDAScore, which were defined as AI scores, or by KIDScore D5 (version 3.2) following the manufacturer’s protocols. A single blastocyst without aneuploidy was transferred after examining the embryonic ploidy status using a next-generation sequencing-based PGT-A platform. Logistic regression analysis with generalized estimating equations was conducted to assess whether AI scores are associated with the probability of live birth (LB) while considering confounding factors.

**Results:**

Logistic regression analysis revealed that AI score was significantly associated with LB probability (adjusted odds ratio [OR] = 2.037, 95% confidence interval [CI]: 1.632–2.542) when pulsatility index (PI) level and types of chromosomal abnormalities were controlled. Blastocysts were divided into quartiles in accordance with their AI score (group 1: 3.0–7.8; group 2: 7.9–8.6; group 3: 8.7–8.9; and group 4: 9.0–9.5). Group 1 had a lower LB rate (34.6% vs. 59.8–72.3%) and a higher rate of pregnancy loss (26% vs. 4.7–8.9%) compared with the other groups (*p* < 0.05). The receiver operating characteristic curve analysis verified that the iDAScore had a significant but limited ability to predict LB (area under the curve [AUC] = 0.64); this ability was significantly weaker than that of the combination of iDAScore, type of chromosomal abnormalities, and PI level (AUC = 0.67). In the comparison of the LB groups with the non-LB groups, the AI scores were significantly lower in the non-LB groups, both for euploid (median: 8.6 vs. 8.8) and mosaic (median: 8.0 vs. 8.6) SETs.

**Conclusions:**

Although its predictive ability can be further enhanced, the AI score was significantly associated with LB probability in SET cycles. Euploid or mosaic blastocysts with low AI scores (≤ 7.8) were associated with a lower LB rate, indicating the potential of this annotation-free AI system as a decision-support tool for deselecting embryos with poor pregnancy outcomes following PGT-A.

**Supplementary Information:**

The online version contains supplementary material available at 10.1186/s12958-024-01185-y.

## Introduction

The concept of morphokinetics has been integrated into the clinical practice of in vitro fertilization (IVF) through ongoing and in-depth evaluations of embryonic development by using time-lapse (TL) monitoring [1, 2]. Morphokinetics involves mapping the developmental profiles of cell divisions and morphological changes (e.g., embryo compaction, blastulation, and the formation of the inner cell mass [ICM] and the trophectoderm [TE]) [3]. Studies have developed several commercial algorithms (e.g., KIDScore D3 and KIDScore D5) for predicting developmental potential or pregnancy outcomes in IVF on the basis of the morphokinetic characteristics of individual embryos and found them to be capable in wide-ranging clinical situations [4–8]. However, time- and labor-intensive manual annotation for morphokinetic parameters may be required before KIDScore algorithms can be employed.

Noninvasive evaluation of blastocyst images by using artificial intelligence (AI) has been introduced in the IVF field to reduce the labor-intensive nature of predicting postimplantation embryo viability. These AI algorithms, which are derived from deep-learning techniques, have been developed to fully automate embryo assessments and eliminate the bias associated with manual evaluation [9–17]. Moreover, the joint use of AI algorithms and TL imaging technology enables analysis of unique embryonic characteristics (e.g., morphokinetic and morphological features) at specific and uniform time points. For example, Bormann et al. collected TL images of blastocysts at 113 h postinsemination (hpi) for training of a CNN model. For implantation prediction, the AI system achieved an AUC of 0.77 and an accuracy of 82.8%, which exceeded that of manual-based embryo selection [11]. Bori et al. employed the combined TL-evaluated morphokinetic and morphological features of individual embryos as input data to predict implantation potential. The implantation prediction accuracy of that AI model, which was trained using an artificial neural network, achieved an AUC of 0.77 [14]. Therefore, knowledge obtained from TL images may provide consistent and informative inputs for the development of AI algorithms.

An AI system called IVY, which was developed using a deep learning model with whole TL videos rather than the TL images, has demonstrated an excellent ability to predict pregnancy with a fetal heartbeat. IVY can analyze and extract embryonic information from entire blastocyst cultivation videos without needing annotated parameters [15]. Berntsen et al. employed a strategy similar to IVY to develop an embryo selection AI model with robustness and generalizability called iDAScore (v1.0). That model was trained using entire sequences of TL images and employed inflated 3D CNN and bidirectional long short-term memory models [17]. The iDAScore training process involved the collaboration of 18 fertility centers with varying IVF protocols; these centers contributed a dataset of more than 14,000 embryos, each associated with known implantation data. This approach was employed to ensure the applicability of the AI system in diverse clinical situations. Several studies have revealed that iDAScore outperforms KIDScore and the Gardner criteria as a model for predicting pregnancy in cycles without preimplantation genetic testing for aneuploidy (PGT-A) [18, 19]. However, the efficacy of iDAScore in cycles with PGT-A remains relatively unexplored.

Our previous study, which employed a high-resolution next-generation sequencing (hr-NGS) platform for PGT-A, revealed that the grading of euploid blastocysts influences both implantation and clinical pregnancy (CP) rates [20]. Moreover, healthy babies can develop from mosaic ETs, but the live birth (LB) rate may decline with an increase in the percentage of aneuploid cells or mosaic complexity [21, 22]. The present study aimed to explore the ability of iDAScore to predict LB outcomes in SET cycles involving euploid or mosaic blastocysts. To achieve this objective, we analyzed the association between LB probabilities of elective SETs and the iDAScore output, considering potential confounders related to clinical outcomes following frozen embryo transfers. These confounders included the IVF cycle characteristics (i.e., patient age, body mass index [BMI], oocyte source, endometrial preparation methods, and serum hormone levels), uterine environment (i.e., endometrial thickness and artery blood flow indices), and ploidy status (i.e., mosaic levels, affected chromosome site numbers, and types of chromosomal abnormalities). In addition, this study compared the clinical outcomes of different iDAScore groups.

## Materials and methods

### Study design and patient selection

This retrospective cohort study was conducted in accordance with the relevant guidelines and regulations. The Institutional Review Board of Chung Shan Medical University approved the study protocol, which was granted a waiver regarding written informed consent (approval number CS1-21156). This study collected IVF data from Lee Women’s Hospital on 426 women undergoing 482 SETs of a frozen–thawed euploid or mosaic blastocyst between January 2017 and June 2021. Patients were excluded if they had an endometrial thickness less than 8 mm, severe endometriosis, or uterine abnormalities (i.e., adenomyosis and congenital or acquired uterine abnormalities). Patients with recurrent implantation failures following PGT-A (ET failures of ≥ 2) were also excluded.

### Embryo culture and evaluations

Laboratory procedures and TL observations were implemented in accordance with the standard protocols described in our previous studies [20, 23]. Briefly, oocytes were collected through controlled ovarian hyperstimulation with either the gonadotrophin-releasing hormone (GnRH) agonist long protocol (Lupron; Takeda Chemical Industries, Osaka, Japan) or the GnRH antagonist protocol (Cetrotide; Merck Serono, Geneva, Switzerland). Mature oocytes were retrieved using ultrasound-guided ovum pickup 36 h after the administration of human chorionic gonadotropin (250 µg, Ovidrel; Merck Serono, Modugno, Italy). Once the oocytes had been fertilized through intracytoplasmic sperm injection or conventional insemination, they were cultured in an EmbryoScope + incubator (Vitrolife, Kungsbacka, Sweden) with a sequential culture system (SAGE Biopharma, Bedminster, NJ, USA). A hypoxic environment containing 6% O_2_, 5% CO_2_, and 89% N_2_ at 37 °C was used for in vitro cultivation. At 118 hpi, individual embryos were annotated in terms for TL morphokinetics, cleavage dysmorphisms, and blastocyst morphology by using EmbryoViewer software (Vitrolife, Kungsbacka, Sweden). The scores of blastocysts generated by KIDScore D5 (version 3.2) or iDAScore ( version 1.0) were collected in accordance with the manufacturer’s protocols (Vitrolife, Kungsbacka, Sweden). The scores obtained from iDAScore, an AI-based scoring system, were referred to as AI scores in this study. Consistent with our previous study [23], this study assigned blastocysts a score from 1 to 7 on the basis of their expansion level. The ICM and TE were also assigned scores ranging from 0 to 2. Blastocyst morphological scores were then calculated using the following formula: expansion score + (ICM score × TE score).

### Next-generation sequencing for PGT-A

D5 or day 6 (D6) blastocysts with diameter ≥ 150 μm and ICM/TE grade > CC (i.e., Gardner embryo grades of AA, AB, BA, BB, AC, CA, BC, and CB) were selected for embryo biopsy. Using micromanipulation techniques, this study isolated five to eight TE cells from individual blastocysts. The isolated cells were thoroughly rinsed with phosphate-buffered saline and then placed on the bottom of a polymerase chain reaction tube that was free from ribonuclease and deoxyribonuclease. The ploidy status of the biopsied blastocysts, including mosaic levels, abnormal chromosome site numbers, and types of chromosomal abnormalities, was determined using the Illumina hr-NGS platform (San Diego, CA, USA). The SurePlex DNA Amplification System (Illumina, USA) and the VeriSeq Preimplantation Genetic Screening Kit (Illumina, USA) were employed to prepare the genomic DNA samples and the DNA libraries from TE cells, respectively, in accordance with the manufacturer’s instructions. Once the individual libraries were normalized and pooled, this study implemented DNA sequencing with a Miseq system, and the sequencing data were analyzed using Bluefuse Multi software (Illumina, USA). The ploidy status of each blastocyst was categorized into the following groups in accordance with the detected level of mosaicism in the biopsied cells: euploid (mosaic level ≤ 20%); low-level mosaic (mosaic level > 20% and < 50%); high-level mosaic (mosaic level ≥ 50% and ≤ 80%); or aneuploid (mosaic level > 80%). Types of chromosomal abnormalities referred to structural patterns of chromosomal abnormalities. Segmental chromosomal alterations referred to mosaic embryos with exclusively segmental abnormalities within the aneuploid compartment. Whole chromosomal alterations referred to mosaic embryos with at least one whole chromosomal abnormality within the aneuploid compartment. Abnormal chromosome site numbers referred to the total number of abnormal segments and chromosomes observed in the aneuploid cells of a mosaic embryo.

### Embryo cryopreservation and transfer

The biopsied blastocysts were incubated for at least 3 h and subsequently cryopreserved. This study employed the Cryotech vitrification method, which involves ultra-rapid freezing and warming techniques (Cryotech, Tokyo, Japan). For each enrolled patient undergoing SET, a blastocyst with euploidy or mosaicism was selected on the basis of its morphological characteristics. Several cycle types were employed to ensure synchronization of endometrial and embryo development. The cycle types included natural, modified natural, and artificial cycles. If patients chose to transfer a mosaic blastocyst, comprehensive counseling was provided by genetic counselors or physicians to inform them about the possible outcomes of mosaic ET and the subsequent procedures to ensure a normal pregnancy. On the day of ET, several measurements were obtained to assess the patient’s condition. The pulsatility index (PI) for uterine artery blood flow and endometrial thickness were evaluated using ultrasonography, and the levels of serum estradiol (E2) and progesterone (P4) were measured. The presence of a visualized intrauterine gestational sac at 5–6 weeks of gestation indicated a CP. A LB was defined as the delivery of a live baby after 24 weeks of gestation. A pregnancy loss (PL) was defined as a CP with the occurrence of a blighted ovum, absence of a fetal heartbeat, intrauterine fetal death or growth restriction, or stillbirth (i.e., fetal death at 20 weeks of gestation or later).

### Statistical analysis

Statistical analyses were performed using GraphPad Prism version 6.0 h (GraphPad Software, San Diego, CA, US) and SPSS Statistics version 26.0 (IBM, Armonk, NY, US). Associations between LB probabilities and the observed variables were analyzed using the generalized estimating equation (GEE) method with both univariate and multivariate logistic regression models. Backward stepwise selection was employed to identify confounding variables (*p* < 0.2) in the dataset. Differences between groups were assessed using the Mann–Whitney U test, chi-squared test, or Fisher’s exact test. Significant trends between groups were determined using analysis of variance or the Cochran–Armitage test. The Spearman correlation test was used to examine the relationships between embryo-related variables. The performance of the LB predictors was evaluated using receiver operating characteristic (ROC) curve analysis. The paired-sample design was implemented using SPSS Statistics to compare two ROC curves in a paired-sample scenario. Statistical significance was indicated at *p* < 0.05 in all analyses.

## Results

### Association between iDAScore and probability of LB

Table 1 presented the patient and cycle characteristics. In total, 364 euploid blastocysts and 118 mosaic blastocysts were included in this study. The associations of the following potential variables with the LB outcomes of elective SETs were analyzed: patient age, anti-Müllerian hormone level, BMI, oocyte source (i.e., autologous or donor oocytes), endometrial preparation method (i.e., artificial cycle, modified natural, or natural cycle), endometrial thickness, PI level (< 3 or ≥ 3), E2 and P4 levels on the day of ET, ploidy status (i.e., euploidy or mosaicism), abnormal chromosome site numbers (0, 1, 2, or > 2), types of chromosomal abnormality (i.e., none, segmental chromosomal alteration, or whole chromosomal alteration), and iDAScore. The results revealed that iDAScore was positively associated with the probability of LB (odds ratio [OR] = 2.002, 95% confidence interval [CI]: 1.607–2.495, *p* < 0.001) in the univariate logistic regression model. Moreover, the PI level and type of chromosomal abnormality were identified as confounding variables when using the backward elimination procedure in the multivariate logistic regression model. The iDAScore was still positively associated with the probability of LB after adjusting for these confounders (adjusted OR = 2.037, 95% CI: 1.632–2.542; *p* < 0.001; Table 2).

**Table 1:** Patient and cycle characteristics

Total SET cycles	482
Female age (years)	36.3 ± 4.9
AMH (ng/mL)	4.4 ± 3.6
BMI (kg/m2)	22.3 ± 3.5
Oocyte sources (%)	
Autologous	420 (87.1)
Donor	62 (12.9)
Endometrial preparation protocols (%)	
Artificial cycles	294 (61.0)
Natural or modified natural cycles	188 (39.0)
Endometrial thickness (mm)	11.6 ± 2.1
Pulsatility index levels (%)	
≥ 3	66 (13.7)
< 3	416 (86.3)
E2 (ET day, pg/mL)	457.7 ± 617.1
P4 (ET day, ng/mL)	38.4 ± 30.5
Ploidy status (%)	
Euploidy	364 (75.5)
Mosaicism	118 (24.5)
Abnormal chromosome site numbers (%)	
0	364 (75.5)
1	72 (14.9)
2	21 (4.4)
> 2	25 (5.2)
Types of chromosomal abnormalities (%)	
None	364 (75.5)
Segmental chromosome alterations	105 (21.8)
Whole chromosome alterations	13 (2.7)
Embryo day (%)	
Day 5	356 (73.9)
Day 6	126 (26.1)
Blastocyst morphological scores	6.4 ± 1.5
Scores of KIDScore D5	6.1 ± 1.7
Artificial intelligence scores of iDAScore	8.4 ± 0.9

**Table 2:** The correlations between the confounding variables and live birth probabilities in this dataset

Variables	Univariate				Multivariate			
	OR	95% CI		*p*	^a^OR	95% CI		*p*
		Lower	Upper			Lower	Upper	
Female age	1.005	0.971	1.040	0.794	–	–	–	–
AMH	0.997	0.955	1.040	0.878	–	–	–	–
BMI	1.014	0.963	1.067	0.599	–	–	–	–
Oocyte sources (autologous vs. donor*)	0.867	0.511	1.473	0.598	–	–	–	–
Endometrial preparation protocols (artificial vs. natural or modified natural *)	0.856	0.599	1.221	0.390	–	–	–	–
Endometrial thickness	1.047	0.961	1.141	0.293	–	–	–	–
Pulsatility index level (< 3 vs. ≥ 3*)	1.389	0.839	2.300	0.202	1.625	0.943	2.800	0.081
Serum E2 level on the ET day	1.000	1.000	1.000	0.642	–	–	–	–
Serum P4 level on the ET day	1.000	0.994	1.006	0.983	–	–	–	–
Ploidy status (euploidy vs. mosaicism*)	1.136	0.741	1.742	0.558	–	–	–	–
Abnormal chromosome site numbers (0 vs. > 2*)	1.967	0.865	4.475	0.107	–	–	–	–
Abnormal chromosome site numbers (1 vs. > 2*)	2.711	1.059	6.944	0.038	–	–	–	–
Abnormal chromosome site numbers (2 vs. > 2*)	0.783	0.241	2.551	0.685	–	–	–	–
Types of chromosomal abnormalities (none vs. whole*)	8.500	1.855	38.946	0.006	6.632	1.382	31.817	0.018
Types of chromosomal abnormalities (segmental vs. whole*)	9.308	1.937	44.728	0.005	9.206	1.817	46.649	0.007
AI scores of iDAScore	2.002	1.607	2.495	< 0.001	2.037	1.632	2.542	< 0.001

The generalized estimating equation (GEE) analysis was used for statistical analysis. The abbreviations “OR”, “^a^OR”,“CI”, “*p*”, “AMH”, “BMI”, “E2”, “P4”, and “AI” denoted odds ratio, adjusted odds ratio, confidence interval, *p* -value, anti-mullerian hormone, body mass index, estradiol, progesterone, and artificial intelligence, respectively. *Indicated of a reference group in the GEE model. The backward stepwise selection was applied to identify the confounders (*P* < 0.2)

### Embryonic and clinical outcomes of blastocysts with stratified AI scores

To compare the differences in embryo quality and clinical outcomes between blastocysts with low AI scores and high AI scores, the blastocysts were divided into quartiles in accordance with the AI scores derived from iDAScore (group 1: AI scores = 3.0–7.8; group 2: AI scores = 7.9–8.6; group 3: AI scores = 8.7–8.9; and group 4: AI scores = 9.0–9.5). The results revealed significant increase trends in the score of KIDScore D5 (from 4.7 ± 1.6 to 7.4 ± 1.1), the blastocyst morphological score (from 5.1 ± 1.2 to 7.4 ± 1.4), and the D5 blastocyst rate (from 23.4 to 100%) as the AI score increased (Table 3). Spearman analysis further verified the significant correlations of iDAScore with KIDScore D5, the blastocyst morphological score, and embryo day (Supplementary Table 1). Moreover, upward trends were also observed in the rates of CP (from 46.7 to 78.8%) and LB (from 34.6 to 72.3%; *p* < 0.05). Bivariate comparisons revealed that group 1 of iDAScore had significantly lower rates of CP (46.7% vs. 62.7–78.8%) and LB (34.6% vs. 59.8–72.3%) and a higher rate of PL (26% vs. 4.7–8.9%) than the other iDAScore groups (Table 3). Moreover, after this study accounted for confounders, SETs with PI level < 3 and blastocysts without whole chromosomal alterations had a higher rate of LB (38.5–69.3%) and a lower rate of PL (6.1–22.2%) compared with SETs with other blastocysts (12.5–56.5% and 16.7–60%) in the iDAScore groups with AI scores < or ≥ 7.9 (Supplementary Fig. 1B).

**Table 3:** The embryonic and clinical outcomes of blastocysts with quartile AI scores

iDAScore	Group 1 (3.0–7.8, *n* = 107)	Group 2 (7.9–8.6, *n* = 136)	Group 3 (8.7– 8.9, *n* = 102)	Group 4 (9.0–9.5, *n* = 137)	Trend tests, *p*
KIDScore D5, mean ± SD	4.7 ± 1.6^abc^	5.7 ± 1.5^ade^	6.4 ± 0.9^bdf^	7.4 ± 1.1^cef^	< 0.001
Blastocyst morphological scores, mean ± SD	5.1 ± 1.2^abc^	6.1 ± 1.4^ade^	6.7 ± 1.1^bdf^	7.4 ± 1.4^cef^	< 0.001
D5 blastocyst, % (n)	23.4% (25)^abc^	69.1% (94)^ade^	98.0% (100)^bd^	100% (137)^ce^	< 0.001
Clinical pregnancy, % (n)	46.7% (50)^abc^	74.3% (101)^a^	62.7% (64)^bd^	78.8% (108)^cd^	< 0.001
Live birth, % (n)	34.6% (37)^abc^	67.6% (92)^a^	59.8% (61)^b^	72.3% (99)^c^	< 0.001
Pregnancy loss, % (n)	26.0% (13)^abc^	8.9% (9)^a^	4.7% (3)^b^	8.3% (9)^c^	0.007

The abbreviations “*p*”, “AI”, “D5”, SD, and “n” denoted *p*-value, artificial intelligence, day 5, standard deviation, and number, respectively. Trends tests were performed by analysis of variance or Cochran–Armitage test. ^a^, ^b^, ^c^, ^d^, ^e^, ^f^ indicates the significant difference (*p* < 0.05) between groups by using the Mann-Whitney U test or the Fisher exact test

### Ability of iDAScore to predict LB probability in SET cycles following PGT-A

**Fig. 1:**
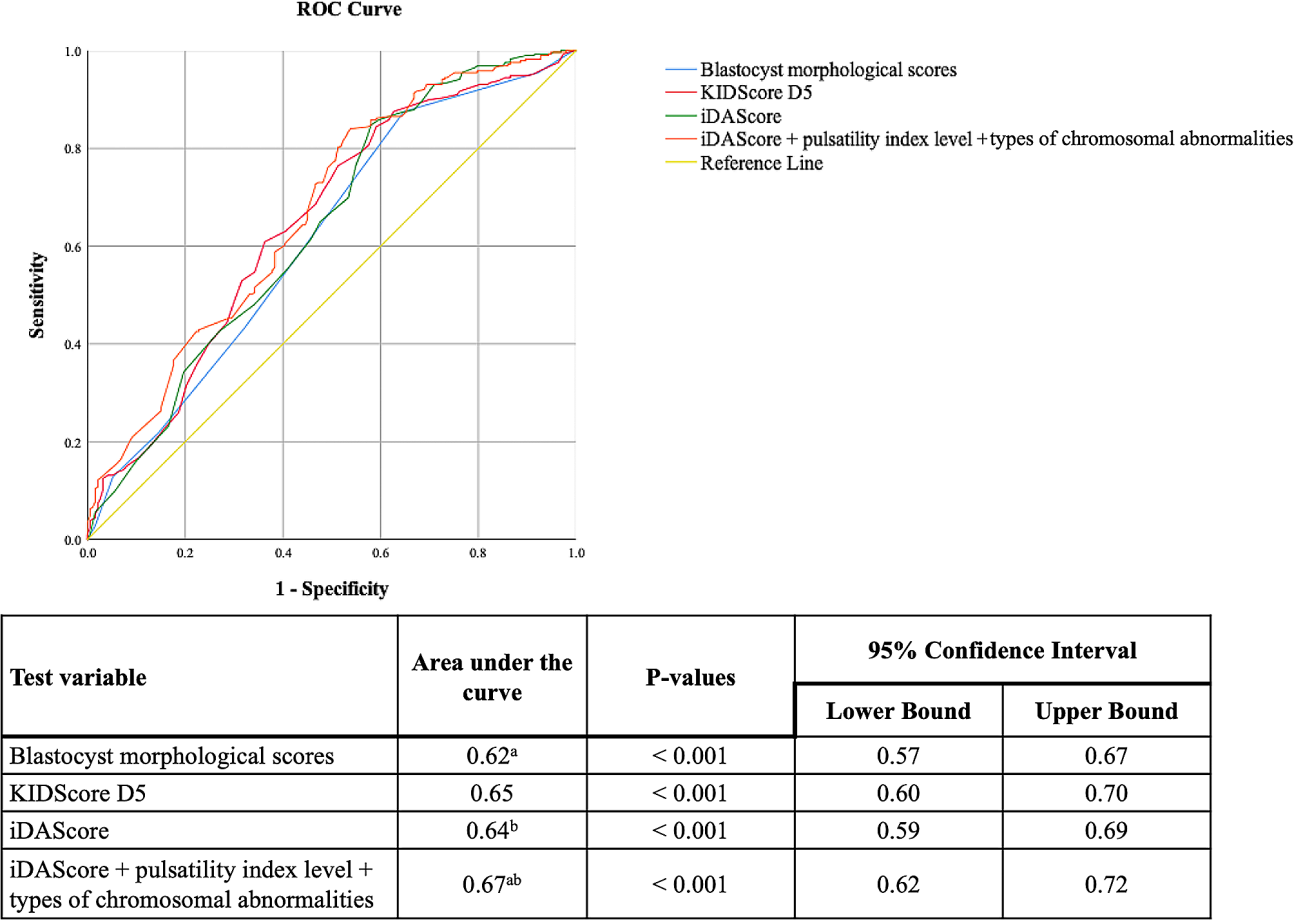
Receiver operating characteristic curve analysis of live birth. The blue, red, green, orange, and yellow curves represented the discrimination of blastocyte morphological scores, KIDScore D5, iDAScore, and the combination of iDAScore, pulsatility index levels, and types of chromosomal abnormalities upon a live birth of the euploid or mosiac SET, respectively. The paired-sample design was applied to compare two areas under the curve (AUCs) for live births. The AUCs for blastocyst morphological scores, KIDScore D5, and iDAScore were found to be similar (0.62–0.65). A significantly increased AUC (0.67) for live birth prediction was obtained by the combination of iDAScore, pulsatility index levels, and types of chromosomal abnormalities. ^a,b^ The same letters denoted significant paired-sample area differences under the ROC curves (*p* < 0.05)

**Fig. 2:**
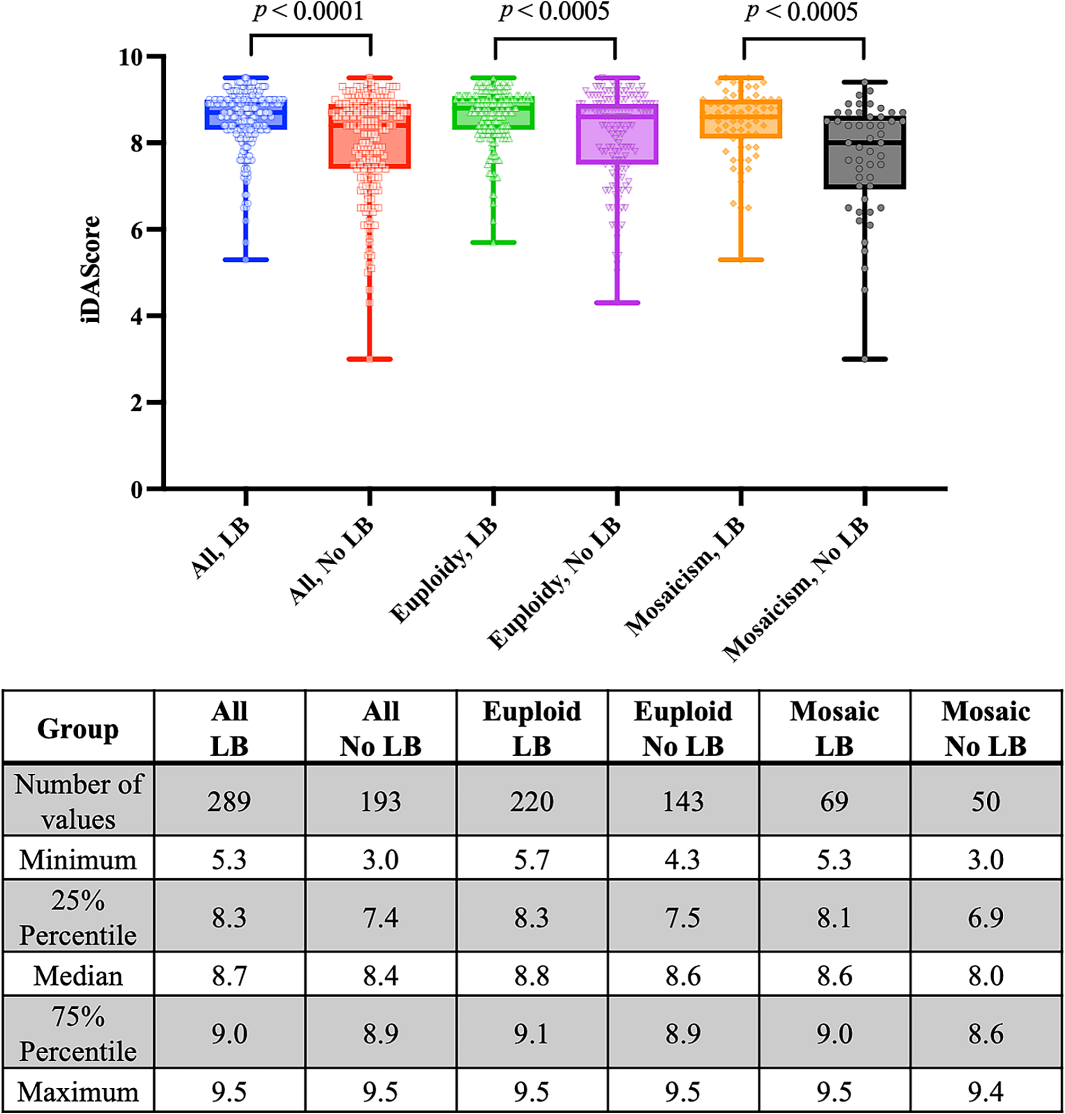
The differences of iDAScore between live birth and non-live birth groups. The live birth groups had higher scores of iDAScore than the non-live birth groups in all SETs (median 8.7 versus 8.4, *p* < 0.0001), euploid SETs (median 8.8 versus 8.6, *p* < 0.0005), or mosaic SETs (median 8.6 versus 8.0, *p* < 0.0005). The significant differences between groups were determined by using the Mann-Whitney U test

The embryos were retrospectively evaluated using TL monitoring to determine the scores for blastocyst morphology, KIDScore D5, and iDAScore. ROC curve analysis was implemented to evaluate the abilities of blastocyst morphology scores, KIDScore D5, and iDAScore to predict LB probability (Fig. 1). Additionally, this study calculated the AUC for LB by considering the combination of three important variables, namely iDAScore, PI level, and the type of chromosomal abnormality. The AUC of iDAScore was 0.64, which was similar to those of blastocyst morphological scores (AUC = 0.62) and KIDScore D5 (AUC = 0.65; *p* > 0.05). Moreover, the combination of iDAScore, PI level, and type of chromosomal abnormality yielded a significantly higher AUC (0.67) for LB compared with the AUCs for blastocyst morphological scores and iDAScore. Although significant statistical results were revealed in ROC curve analysis (Fig. 1), the AUCs of the analyzed methods were still less than 0.7, suggesting that their prediction abilities for LB in PGT-A cycles remained limited. Nevertheless, when comparing the LB and non-LB groups, the AI scores differed not only in euploid SETs (median: 8.8 vs. 8.6; *p* < 0.0005) but also in mosaic SETs (median: 8.6 vs. 8.0; *p* < 0.0005; Fig. 2).

## Discussion

The use of hr-NGS for PGT-A enables the analysis of all 23 pairs of chromosomes, with sensitivity to the level of 10 Mb in size. This high resolution enables a detailed evaluation of embryonic ploidy characteristics and helps distinguish between euploidy and aneuploidy, facilitating the efficient identification of mosaicism, as well as whole or segmental chromosomal alterations and abnormal chromosome site numbers [22, 24]. Clinical investigations have revealed that compared with array comparative genomic hybridization, hr-NGS provides more precise identification of euploid embryos, leading to improved pregnancy outcomes in single-euploid ET cycles [25, 26]. In addition, our research supports the notion that mosaic embryos can, especially for patients without any available euploid embryos, be considered for transfer, albeit with caution regarding potential risks and undesired negative effects. Similar to previous reports, the results indicate that types of embryonic mosaicism may affect clinical outcomes in mosaic ET cycles [22], but most LB babies are healthy and have a low risk of abnormal ploidy [21, 27–29]. Moreover, advanced embryo analysis software accompanied by TL annotations can be used to effectively rank euploid blastocysts by their implantation potential [20, 30]. This study further confirmed the effectiveness of an annotation-free AI model (iDAScore) for blastocyst ranking in both euploid and mosaic SETs by investigating the predictive capability of AI scores and the associations of AI scores with LB probabilities.

Consistent with the findings of other studies, our findings indicate that characteristics of embryonic mosaicism affect the LB rates of SETs following PGT-A [21, 22]. Viotti et al. revealed that the crucial characteristics of mosaicism that significantly affect clinical outcomes were the mosaic level (< 50% vs. ≥ 50%), type of chromosomal abnormalities (i.e., segmental vs. whole), and aberrant chromosome numbers (≤ 2 vs. > 2). The abortion rate of implanted embryos with whole chromosomal alterations (25%) was significantly higher than that of euploid embryos (8.6%). In addition, embryos with complex mosaicism (i.e., abnormal chromosome numbers > 2) exhibited the lowest ongoing pregnancy and birth rate (20.8%) [22]. This study followed the prioritization criteria for embryo transfer outlined in our previous publication [22] and enrolled the cycles with SET following PGT-A. As a result, 24.5% (*n* = 118) of transferred blastocysts were identified as mosaic embryos, while only 2.7% (*n* = 13) of transferred blastocysts were found to have whole chromosome alternations (Table 1). In this scenario, the univariate logistic regression analysis in this study indicated a nonsignificant association between embryonic mosaicism and LB when compared with embryonic euploidy. A possible explanation for this result is that most of the mosaic embryos in this study had low-level mosaicism (83.1%, 98/118) with only one segmental abnormality (65.3%, 64/98). Additionally, the sample sizes of high-level mosaic embryos (16.9%, 20/118), embryos with whole chromosomal alterations (11%, 13/118), and embryos with more than two abnormal chromosomal sites (21.2%, 25/118) were too small to present the adverse effects of embryonic mosaicism on IVF outcomes. Nevertheless, alterations to chromosomal structures appeared to substantially affect LB probability in this study. The backward stepwise selection in the multivariate logistic regression model revealed significantly increased LB probability in embryos without chromosomal abnormalities (adjusted OR = 6.632) and in embryos with segmental chromosomal alterations (adjusted OR = 9.206) compared to embryos with whole chromosomal alterations (Table 2).

The backward stepwise selection in the logistic regression model also revealed that impedance to uterine artery blood flow (i.e., the PI level) was a confounder in this dataset. Studies have described the importance of uterine PI levels that were measured using Doppler ultrasound investigations in IVF–ET cycles [31–34]. One study revealed a positive correlation between IVF outcomes and the uterine scoring system for reproduction, which incorporates the PI level, endometrial thickness, endometrial layering, endometrial motion, endometrial blood flow in zone 3, and myometrial blood flow [34]. Steer et al. and Cacciatore et al. asserted that PI level > 3.0 on the day of ET was associated with low uterine receptivity, and implantation became unachievable when the PI levels were > 3.3 [31, 32]. Similarly, the results of this study revealed an adjusted OR of 1.625 (*p* = 0.081) for LB when comparing the group with PI level < 3 and the group with PI level ≥ 3 (Table 2). However, the clinical outcomes in the group with PI level < 3 were only slightly better than those in the group with PI level ≥ 3 (*p* > 0.05; Supplementary Fig. 1A).

With the ultimate goal of facilitating a normal singleton-term pregnancy through assisted reproductive technology, this study assessed the predictive ability of iDAScore with respect to euploid or mosaic embryo selection. Ueno et al. have validated the performance of iDAScore in non-PGT-A cycles, and this model had a better ability to predict CP than traditional assessments of blastocyst morphology, especially in patients younger than 35 years (AUC = 0.72) [18]. Blastocysts with greater iDAScore had an increased rate of LB and decreased rate of PL [19]. However, few studies have examined the efficacy of iDAScore following PGT-A. Cimadomo et al. implemented a retrospective study to externally validate the efficacy of iDAScore in 808 euploid transfers. Although the PGT-A platform used in that study could only discriminate between euploid and aneuploid embryos, logistic regression revealed that iDAScore was positively associated with LB (OR = 1.30), without considering potential confounders. The iDAScore achieved an AUC of 0.66 for LB prediction, which was comparable to the performance of embryologists (AUC = 0.64) [35]. With continual advancements in technology, hr-NGS offers a superior ability to detect chromosomal mosaicism in IVF embryos. The present study validated the positive and significant associations between iDAScore and LB probability following euploid or mosaic SETs. Each unit increase in the iDAScore was associated with greater odds of achieving a LB not only in the univariate model (2.002 times) but also in the multivariate model (2.037 times), which adjusted for the confounders related to uterine environment and ploidy status (Table 2). The predictive ability of iDAScore (AUC = 0.64) was comparable to that of the annotation-required KIDScore D5 (AUC = 0.65) and blastocyst morphological score (AUC = 0.62), as revealed through uniform time-point assessments (Fig. 1). Importantly, this study revealed that combining iDAScore with PI levels and types of chromosomal abnormalities significantly improved the AUC for predicting LB to 0.67 (Fig. 1 and Supplementary Fig. 1B). Moreover, a comparison of the AI scores between SETs with LB and without LB verified that AI scores were significantly higher in the LB groups of both euploid (median: 8.8 vs. 8.6) and mosaic SETs (median: 8.6 vs. 8.0; Fig. 2).

As mentioned in the literature review, the embryonic features related to failed implantation significantly decrease the odds of a successful LB following PGT-A; they include poor blastocyst morphology, unfavorable ICM or TE morphology (i.e., grade C), low blastocyst quality (i.e., < BB), and delayed blastocyst formation (i.e., biopsied on day 6 or 7) [36]. Our previous study successfully converted blastocyst morphological components (i.e., expansion levels, ICM grades, and TE grades) into TL-based numeric blastocyst morphological scores [23]. The present study revealed that the blastocysts in the low AI score group (scores 3.0–7.8) had a low blastocyst morphological score (5.1 ± 1.2) and a low D5 blastocyst rate (23.4%; Table 3), which were negatively associated with LB probability (Supplementary Table 2). In addition, the Spearman correlation analysis verified that the iDAScore was significantly correlated with blastocyst morphological score and embryo day of transferred blastocysts (Supplementary Table 1). These results were similar to those reported by Cimadomo et al., who demonstrated that the iDAScore yielded better results in blastocysts with rapid development and good morphological quality in the biopsied blastocysts [35], and provided one potential explanation for the positive association between iDAScore and LB probability. In addition, Ezoe et al. attempted to uncover the inner workings of deep learning–based iDAScore by collecting and evaluating TL information. They asserted that morphogenetic features—such as irregular first division and prolonged time intervals during embryonic cleavages, compaction, and blastulation—were negatively associated with AI scores [37]. These aberrant morphokinetic features have been demonstrated to negatively affect the pregnancy outcomes of euploid ETs in one study [36]. Notably, these morphogenetic features were also found to be key components of KIDScore D5 [20]. In accordance with the aforementioned results, the current study revealed a strong correlation between iDAScore and KIDScore D5 (Spearman correlation coefficient: 0.731; Table 3 and Supplementary Table 1), resulting in similar abilities to predict LB (AUC = 0.64–0.65; Fig. 1).

The primary limitation of this single-center study was its retrospective nature, which may have led to a lack of randomization and resulted in selection bias. Randomized controlled trials are thus required to assess the clinical value of iDAScore. Multiple SETs from several couples were present in the dataset, which may have introduced bias to the estimation of regression parameters. Therefore, this study implemented the GEE method to analyze repeated measurements. The GEE is a well-known method for longitudinal data analysis that addresses potential intrasubject correlations [38]. Additionally, the sample size (2.7%, 13/482) of embryos with AI scores < 6 was small, given the criteria for embryo biopsy and PGT-A. The dataset also exhibited a skewed distribution of embryo data, with a concentration of AI scores ≥ 8.0 (75.3%, 363/482), which may have led to an underestimation of the predictive ability of iDAScore. Although this study, along with our previous study [21], confirmed that healthy live births could be delivered from mosaic embryo transfers, these results should be interpreted with caution because several studies have revealed that embryonic mosaicism can persist during pregnancy, leading to the development of mosaic fetuses [29] and even babies with mosaicism [28]. Therefore, patients should receive adequate and comprehensive genetic counseling before ET on the possible outcomes of transferring mosaic embryos. Although no cases of placental or fetal mosaicism were identified in this study, a standardized approach for verifying fetal mosaicism during pregnancy is essential to ensure a normal pregnancy. With respect to generalizability, this study revealed the iDAScore might be applicable for ranking the euploid or mosaic embryos in our clinical setting. However, these results must be interpreted cautiously regarding the patient selection criteria. Patients with thin endometrial thickness, uterine abnormalities, and recurrent implantation failures were excluded from this study. Hence, the results might not represent all patients who underwent IVF treatment.

## Conclusion

Although PGT-A offers promising clinical outcomes, the overall pregnancy rates of euploid SETs usually do not exceed 50–60%, implying differences in developmental potential among genetically screened blastocysts. Establishing noninvasive tools for automated embryo assessment is thus preferable for standardizing IVF protocols and shortening the time to pregnancy. The predictive ability of iDAScore can be further enhanced; however, in consideration of the maternal and chromosomal confounders, this study concludes that AI scores are significantly associated with LB probabilities following PGT-A. Therefore, this annotation-free AI system constitutes a potential decision-support tool for deselecting unfavorable embryos in euploid or mosaic SET cycles.

### Electronic supplementary material

Below is the link to the electronic supplementary material.

Supplementary Material 1

**Supplementary Fig. 1**. Comparison of clinical pregnancy (CP), live birth (LB), and pregnancy loss (PL) rates in SETs with different pulsatility index levels (**A**) and combinations of iDAScore, pulsatility index levels, and types of chromosomal abnormalities (**B**) following preimplantation genetic tests for aneuploidy. Clinical results of the group with pulsatility index levels < 3.0 were better but not significant than the group with pulsatility index levels ≥ 3.0. Moreover, the clinical outcomes of the blastocysts with pulsatility index levels < 3.0 and non-whole chromosome alterations were better than the rest blastocysts in both iDAScore < 7.9 and ≥ 7.9 groups. The differences between groups were determined by the Fisher exact test or the chi-square test

## Data Availability

The datasets generated during the current study are available from the corresponding author on reasonable request.
