# Supplementary material for: Associations between the artificial intelligence scoring system and live birth outcomes in preimplantation genetic testing for aneuploidy cycles
Source: Reprod Biol Endocrinol. 2024 Jan 17;22:12. doi: 10.1186/s12958-024-01185-y (PMC10792866; doi:10.1186/s12958-024-01185-y)
Supplement: Supplementary file 1 — Supplementary Material 1 [file 12958_2024_1185_MOESM1_ESM.docx]

Supplementary Table 1. Spearman correlation analysis of embryonic variables.

| Factors | iDAScore | KIDScore D5 | Blastocyst morphological scores | Embryo day  (D6 or D5) |
| --- | --- | --- | --- | --- |
| iDAScore | 1.000 | 0.731^*^ | 0.592^*^ | -0.670^*^ |
| KIDScore D5 | 0.731^*^ | 1.000 | 0.703^*^ | -0.662^*^ |
| Blastocyst morphological scores | 0.592^*^ | 0.703^*^ | 1.000 | -0.519^*^ |
| Embryo day (D6 or D5) | -0.670^*^ | -0.662^*^ | -0.519^*^ | 1.000 |

The abbreviations “D5” and “D6” denoted day 5 and day 6, respectively. *Indication of significant correlations between groups.

Supplementary Table 2. The correlations between the embryonic variables and live birth probabilities.

| **Variables** | **Univariate** | | | | **Multivariate** | | | |
| --- | --- | --- | --- | --- | --- | --- | --- | --- |
|  | **OR** | **95% CI** | | ***p*** | **^a^OR** | **95% CI** | | ***p*** |
|  |  | **Lower** | **Upper** |  |  | **Lower** | **Upper** |  |
| KIDScore D5 | 1.399 | 1.237 | 1.582 | < 0.001 | 1.413 | 1.246 | 1.603 | < 0.001 |
| Blastocyst morphological scores | 1.302 | 1.136 | 1.492 | < 0.001 | 1.306 | 1.139 | 1.498 | < 0.001 |
| Embryo day  (D5 vs. D6*) | 3.105 | 2.018 | 4.777 | < 0.001 | 3.272 | 2.087 | 5.130 | < 0.001 |

The generalized estimating equation (GEE) analysis was used for statistical analysis. The abbreviations “OR”, “^a^OR”, “CI”, “*p*”, “D5”, and “D6” denoted odds ratio, adjusted odds ratio, confidence interval, *p*-value, day 5, and day 6, respectively. *Indication of a reference group in the GEE model. Confounding variables, i.e., pulsatility index levels and types of chromosomal abnormalities, were used for adjustment in the multivariable logistic regression analysis.
