# Supplementary figures and images for: Associations between the artificial intelligence scoring system and live birth outcomes in preimplantation genetic testing for aneuploidy cycles
Source: Reprod Biol Endocrinol. 2024 Jan 17;22:12. doi: 10.1186/s12958-024-01185-y (PMC10792866; doi:10.1186/s12958-024-01185-y)

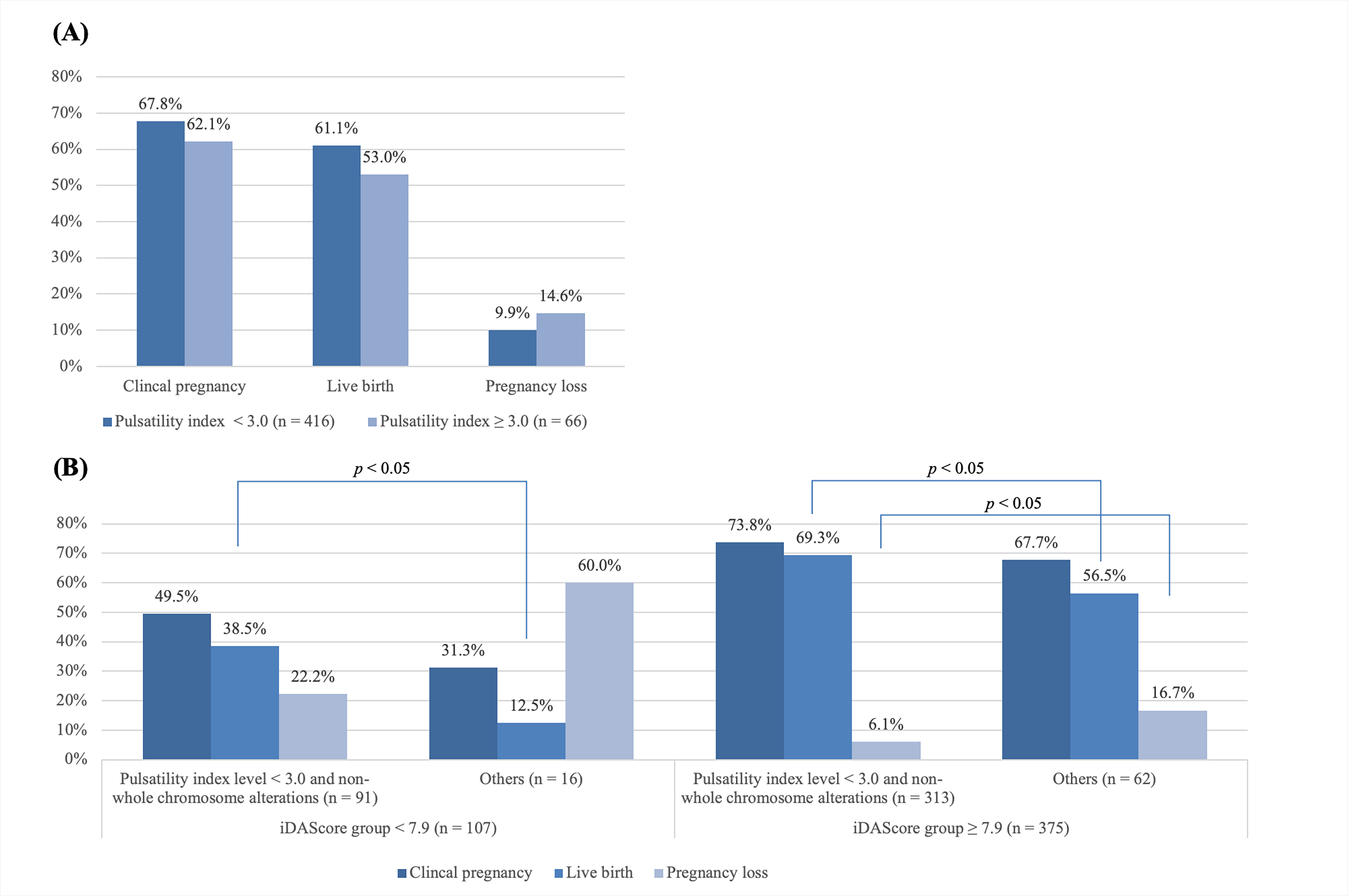

Supplement: Supplementary file 2 — Supplementary Fig. 1. Comparison of clinical pregnancy (CP), live birth (LB), and pregnancy loss (PL) rates in SETs with different pulsatility index levels (A) and combinations of iDAScore, pulsatility index levels, and types of chromosomal abnormalities (B) following preimplantation genetic tests for aneuploidy. Clinical results of the group with pulsatility index levels < 3.0 were better but not significant than the group with pulsatility index levels ≥ 3.0. Moreover, the clinical outcomes of the blastocysts with pulsatility index levels < 3.0 and non-whole chromosome alterations were better than the rest blastocysts in both iDAScore < 7.9 and ≥ 7.9 groups. The differences between groups were determined by the Fisher exact test or the chi-square test [file 12958_2024_1185_MOESM2_ESM.tiff]
